# Higher incidence of hematuria was observed in female children with microtia

**DOI:** 10.1038/s41598-023-41330-y

**Published:** 2023-09-11

**Authors:** Na Sun, Yang Yang, Fengli Jiang, Yuanyuan Wu, Bo Pan, Sien Zhan

**Affiliations:** Plastic Surgery Hospital, Chinese Academy of Medical Sciences, Peking Union Medical College, Beijing, 100144 China

**Keywords:** Biomarkers, Paediatrics, Orthopaedics

## Abstract

The goals of this study were to investigate the incidence and characteristics of hematuria in patients with microtia, and to clarify that more attention should be paid to renal dysfunction in patients with microtia. We conducted a retrospective cohort study of a total 9447 children diagnosed with microtia (selected as study group, 7037 children) or pigmented nevus (selected as control group, 2410 children) at the Plastic Surgery Hospital, Chinese Academy of Medical Sciences and Peking Union Medical College from January 2009 to June 2021. All of the routine urinalysis report of these children were reviewed to assess the incidence and characteristics of hematuria in each group. No statistically significant differences were observed when analyzing the overall incidence of hematuria between the study and control groups (*P* > 0.05). However, after grouping by sex, the incidence of hematuria in female children with microtia was significantly higher than that in femalecontrol group and no similar results were observed in the male patients. In addition, after further grouping by age in case group, the incidence of hematuria in girls of all ages with microtia was significantly higher than that in males with microtia (age 0–10:males: Girls = 1.89%:4.14%; age 0–5: males: Girls = 1.22%:3.73%; age 6–10: males:Girls = 1.97%:4.14%,P < 0.05), while no similar results were obtained in the control group.(age 0–10:males: Girls = 1.39%:2.22%; age 0–5: males: Girls = 1.07%:1.95%; age 6–10: males: Girls = 3.38%:3.17%, *P* > 0.05). Higher incidence of hematuria was observed in female children with microtia.

## Introduction

Microtia is a congenital malformation associated with abnormal migration of neural crest cell in the first and second branchial arch during the embryonic stage^1,2^. The presentation of the auricular deformity can range from mild structural anormality to severe absence of the ear and often accompanied by hearing loss. As the second most common congenital craniofacial condition after cleft lip^2^, reported prevalence of the microtia ranges from 0.83 to 17.4 per 10,000 births depending on race^3,4^, region^5,6^, sex, and even altitude^3^. Although microtia commonly occurs as an isolated anomaly it can also present as a component of some specific congenital malformations or syndromes such as Goldenhar, Treacher-Collins, Branchio-oto-renal, Nager syndromes and others^7^. Therefore, microtia is known to combine with other organ anomalies.

Urinary anomalies, according to the report, occur 19 times more likely in microtia patients comparing with general population^8^. These includes various renal abnormalities^9^, such as horseshoe kidney, vesicoureteral reflux, renal agenesis, and so on. Sameer et al. also found that patients with both isolated and syndromic microtia may be at risk for renal anomalies which may not be undetected at birth^10^. Therefore, renal ultrasound screening was not only recommended in patients with ear anomalies accompanied dysmorphic features, but also in non-syndromic patients ^11^. However, there was no clear guidelines concerning an appropriate urinary screening protocol for patients with microtia^11–15^. In addition, most previous studies focused on the urological structural abnormalities in microtia patients. There is little attention has been paid to urinary functional abnormalities. Compared with structural studies, functional studies are equally important and perhaps more meaningful for the early detection of diseases of an organ or system. Therefore, more attention should be paid to functional screening of the urinary system in patients presenting with microtia.

Hematuria, including macroscopic hematuria and microscopic hematuria, is one of the most important signs of urinary functional abnormalities and may indicate the occurrence of asymptomatic urinary disease at an early stage^16^. Hence, the present study was going to identify the incidence and characteristics of hematuria in patients with microtia.

## Methods

### Study population

We retrospectively enrolled microtia patients and pigmented nevus patients aged between 0 and 10 years old who were received treatment in Plastic Surgery Hospital of Peking Union Medical College from January 2009 to June 2021.Patients with definite causes of hematuria such as recent trauma, strenuous exercise, menstruation, metabolic and pharmacologic factors (beets, melanin, bile, porphyrin, iron, and rifampicin), microtia patients without routine urinalysis reports were excluded in our research. All of the routine urinalysis reports of these children were analyzed to evaluated the incidence and characteristics of hematuria in the study population. The subjects were divided into two groups: the case group (microtia group) and the control group (pigmented nevus group). According to the patient’s sex and age, the patients were also grouped into the following subgroups: male/femalegroup, 0–5 age male/femalegroup, 6–10 age male /femalegroup.

### Urine collection and urinalysis

All the children in the study were asked to collect at least 10 ml of clean voided midstream random urine specimens. For children not toilet trained, urine samples were collected with urine bags. Urine samples were tested by the automatic urine analyzer (UA5800, Shenzhen Mindray Bio-Medical Electronics Co., Ltd., Shenzhen, China). If occult blood test of urine was positive, this sample should be immediately centrifuged at 1500 revolutions per minute for 5 min, discarded the supernatant and suspended the sediment in 0.3 ml of supernatant for microscopic evaluation. Urine samples should be detected within 2 h after collection. In addition, the international quality control and the external quality assessment of the instrument were meet the quality control standard. Hematuria is defined as the presence of ≥ 3 red blood cells per high-power field on microscopic analysis of a properly collected and analyzed urine sample, which is the general defination of microscopic hematuria^17^.

### Statistical analysis

The statistical analysis was performed using STATA 16.0 software. Continuous data variables were described by mean ± standard deviation, and categorical variables was expressed using percentage. The Fisher’s exact test was used for descriptive univariate statistics, whereas the t-test was used for normally distributed data. The two tailed p-values were derived from the calculated test statistics, and *p* < 0.05 was considered significant.

All protocols was approved by the ethics committee of the Plastic Surgery Hospital, Chinese Academy of Medical Sciences.

### Ethical approval

All procedures in this study were conducted in accordance with the Institutional Research Ethics Board of Plastic Surgery Hospital, Chinese Academy of Medical Sciences, Peking Union Medical College (No. 2022-139) approved protocols. Individual consent for this retrospective analysis was waived by Plastic Surgery Hospital, Chinese Academy of Medical Sciences, Peking Union Medical College.

## Results

### The characteristics of the demographics of patients

**Figure 1:**
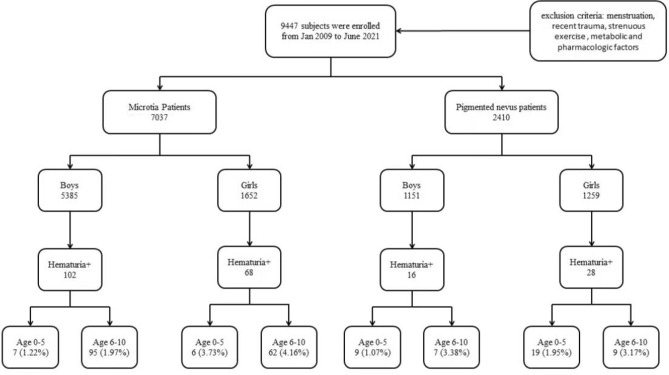
The characteristics of the demographics of patients.

As shown in Fig. 1, a total of 9447 subjects were enrolled in our study from Jan 2009 to June 2021. The microtia group included 7037 children with 5835(82.92%) males and 1652(17.08%) girls, whose mean age was7.24 ± 2.00 and 7.48 ± 1.73 respectively. There were 1151 (47.76%) males and 1259 (52.24%) girls with a mean age of 4.03 ± 2.47 years old in 2410 pigmented nevus patients. No significant difference of age was found between the case and control groups. However, we found significant differences in the sex between two groups (*P* < 0.05).

As shown in Table 1, a significantly higher frequency of hematuria was observed in study group {microtia: pigmented nevus: 2.42% vs 1.83%, *P* = 0.093 (95% CI 0.537–1.050)}, but there is no significant difference was found between the two groups (*p* = 0.093). The prevalence of hematuria in males with microtia and pigmented nevus was 1.89% and 1.39% respectively (*p* = 0.244, 95% CI = 0.274–0.147). In female group, the prevalence of hematuria in patients with microtia was nearly two times higher than that in patients with pigmented nevus (4.12% vs. 2.22%, *p* = 0.005, 95% CI 0.339–0.828). Among the three intergroup comparisons, only the positive rate of hematuria between female microtia group and the femalepigmented nevus group had statistically significant differences (χ^2^ = 8.022, *p* = 0.005), and no similar results were observed in another two intergroup comparison.

**Table 1:** Intergroup comparisons of the incidence of Hematuria.

Age and sex groups	Microtia	Pigmented nevus	*P*	95%CI	OR
All children	7037	2410	–	–	–
Hematuria ( +)	170 (2.42%)	44 (1.83%)	0.093	0.537–1.050	2.956
Girls	1652	1259	–	–	–
Hematuria ( +)	68 (4.12%)	28 (2.22%)	0.005	0.339–0.828	8.324
Males	5385	1151	–	–	–
Hematuria ( +)	102 (1.89%)	16 (1.39%)	0.244	0.274–0.147	1.451

The patients were divided into subgroups according to sex and age in order to further investigate the correlation between the incidence of hematuria and patients (Table 2). The number of males with microtia was significantly higher than that of girls with microtia (5385 vs. 1652). In case group, the incidence of hematuria in girls of all ages with microtia was significantly higher than that in males with microtia (4.12% vs. 1.89%, 3.72% vs. 1.22%, 4.16% vs. 1.97%), which were significantly different among the three age groups (all *p* < 0.05) (Table 3). While no similar results were obtained in the comparison of hematuria positive rate among subgroups of the control group (all *p* > 0.05) (Table 2).

**Table 2:** Comparisons of the incidence of Hematuria between different age and sex groups in pigmented nevus group.

Age and sex groups	Males	Girls	*P*	95%CI	OR
Number (age 0–10), N (%)	1151	1259	–	–	–
Hematuria ( +)	16 (1.39%)	28 (2.22%)	0.127	0.868–2.998	0.571
Number (age 0–5), N (%)	844	975	–	–	–
Hematuria ( +)	9 (1.07%)	19 (1.95%)	0.127	0.830–4.098	0.474
Number (age 6–10), N (%)	207	284	–	–	–
Hematuria ( +)	7 (3.38)	9 (3.17%)	0.017	0.342–2.553	0.778

**Table 3:** Comparisons of the incidence of Hematuria between different age and sex groups in microtia group.

Age and sex groups	Males	Girls	P	95%CI	OR
Number (age 0–10), N (%)	5385	1652	–	–	–
Hematuria ( +)	102 (1.89%)	68 (4.12%)	< 0.001	1.628–3.031	0.456
Number (age 0–5), N (%)	572	161	–	–	–
Hematuria ( +)	7 (1.22%)	6 (3.73%)	0.034	1.480–11.605	0.327
Number (age 6–10), N (%)	4813	1491	–	–	–
Hematuria ( +)	95 (1.97%)	62 (4.16%)	< 0.001	0.135–0.573	0.473

### Auxiliary examination results in microtia patients with positive hematuria

The auxiliary examination results in microtia patients with positive hematuria were reviewed in Table 4. Among the 170 microtia patients with positive hematuria, 158 had electrocardiogram results, 126 underwent radiography inspection, 79 had ultrasonography results. A total of 38 patients with microtia had electrocardiogram abnormality. 3.17% (across the 126 cases, n = 4) were accompanied by abnormalities of radiography, consisting of abnormal development of auditory ossicles (n = 1), aortic regurgitation (n = 1), dense mediastinal shadow (n = 1), scoliosis (n = 1). Among the 79 microtia patients undergoing ultrasonography examine, 1 case had punctate calcification in the right lobe of liver, 1 case had patent foramen ovale, 2 cases had little regurgitation of mitral and tricuspid valves. According to the above examination results, none of the 170 microtia patients with positive hematuria patients was complicated with renal structural abnormalities.

**Table 4:** Auxiliary Examination Results in 170 Microtia Patients with Positive Hematuria.

Auxiliary examination	Total	Abnormality (N, %)
Electrocardiogram	158	38 (24.05%)
Radiography	126	4 (3.17%)
Ultrasonography	79	5 (6.33%)

## Discussion

This retrospective study of a large patient cohort at our hospital serves to investigation of a relationship between microtia and hematuria. In the aspect of demographic data, the cohort was fairly representative of known epidemiologic data of microtia patients. Our population was 76.5% male which was consistent with known epidemiological trends of this patient population in the word^18–22^. The present study suggested the high incidence of hematuria in patients with microtia compared with pigmented nevus patients as control group, esprcially in female children.

Hematuria, as one of the common clinical symptoms related to urinary system, is the most important laboratory signs of renal disease in children^23–27^. In addition, the hematuria aslo played an important role in the diagnosis and treatment of chronic kidney disease and acute kidney injury^28^. Although the vast majority of microscopic hematuria were benign especially in children presenting with isolated asymptomatic microscopic hematuria^29,30^. There are still a proportion of patients could be progressive aggravation, or even progress to end-stage renal disease (ESRD). In addition, Mastaneh had reported that the incidence of ESRD in children with persistent asymptomatic hematuria was nearly 20 times higher than that in children without hematuria^31^.

Several studies demonstrated that the prevalence rate of hematuria in children were 0.59%, 1.1%, 3.86%, respectively in China^32^^.^ In addition, the positive rate of hematuria ranged from 0.1% to 1.2% according to epidemiological investigation on children's hematuria in other countries such as Korea, Japan, Iran and so on^33–38^. In the present study the prevalence rate of hematuria in microtia girls was reported markedly increased comparing with those in above literature. Many factors can affect the prevalence of hematuria, such as examination standard, equipment, operators, age groups of selected children, geographic region, ethnic background etc. As we all known, each study has its own clinical implications. The overall prevalence rate of hematuria in girls was higher than that in males (4.12% vs 1.89%), and the incidence of hematuria in female children was more prevalent compared with males in all age groups. Although some epidemiological studies also found that hematuria had been more prevalent in girls as compared to males, there is no difference was observed in the positive rate of hematuria between males and girls in our control group.

Hematuria has a multitude of causes. While, considering the higher incidence of hematuria coexisted microtia, urological dyplasia should still be considered at first step. The association between ear and urorlogical abnormalities has been long recognized; however, the connection between these two disparate organs is not always straightforward^39^. Although Alport syndrome with auricular and renal manifestation^40^ is the most well-known, there are over 20 disorders that need to be considered in the differential diagnosis of patients with both ear and kidney abnormalities. Commonalities are present between the kidney and ear in a number of structural proteins^41^ developmentally important transcription factors, ciliary proteins, and channelproteins, and mutations in these pathways can lead to disease in both organ systems^39^.

In our study, all the 170 microtia children with positive hematuria underwent ultrasound examination. There is no renal abnormality observed. While, considering the known relationship between microtia and renal anomalies, some hidden kidney structural abnormalities might have been missed by ultrasound. Like glomerular structural abnormalities, thin basement membrane nephropathy caused by collagen chains’ alteration, which is the common cause of microscopic hematuria in children^42^. And its diagnosis is confirmed under electron microscopy with diffuse basement membrane lamellation of bioposy^43^. Besides, in terms of another common urologiccal manifestation accompanied with microtia, vesicoureteral reflux, the accuracy of renal ultrasound diagnosis is low. The sensitivity is only 18–46% if absence of pyelectasis or other modality change^44^.

As known, vesicoureteral reflux is the most frequently detected cause in pediatric recurrent urinary tract infection cases, with an incidence of 30–50%^45^. The primary concern of Vesicoureteral reflux is the risk of recurrent pyelonephritis. Epidemiologically, pyelonephritis is more common in women and girls are outnumbered by males among young infants. This is may be the underlying cause of significantly higher incidence of hematuria in girls observed in the present study. Historically, children with any grade of vesicoureteral reflux were thought to be under substantial risk of permanent renal damage^46^. And early detection and appropriate management of vesicoureteral reflux in children is an important approach to improve renal outcomes^47^.

Therefore, if we only use the renal ultrasound to screen the renal structural abnormalities in patients with microtia, we may delay the optimal timing of their treatment. And due to lack clear guidelines concerning an appropriate renal screening protocol for patients with microtia, not all patients underwent renal ultrasonography in our study. In addition, even if the structure is normal, functional abnormalities cannot be ruled out. The finding of our study prompted that we should not only pay attention to the renal structural abnormalities, but also concern about the functional index sign of renal, which will help us to detect, prevent and treat diseases early.

At present, more and more attention has been paid to the study of asymptomatic hematuria. In Japan, Korea and Taiwan, mass urinary screening programs for children have been established for many years^48^. Urinalysis is a simple screening test for kidney diseases in children, which is very useful, inexpensive, widely available and non-invasive, and easy to be carried out widely in the population. Therefore, we suggest that hematuria can be used as a supplementary test for screening renal congenital malformation in patients with microtia, which will help us to earlier recognition of microtia associated renal anomalies, perform earlier nephrological or urological intervention, and decrease fewer subsequent renal complications.

One strength of our study is that we firstly investigated the incidence and characteristics of hematuria in patients with microtia in China, which is able to provide valuable insights into the incidence of microscopic hematuria in female pediatric microtia patients. In addition, our hospital is a leading authority in auricle reconstruction, attracting most of the country’s microtia patients for treatment. So, the other strength of the study is a relatively a large sample size and the variety of diseases, which can have certain representativeness. However, several limitations of this study should be considered when interpreting the present results. Firstly, this study was a single-center retrospective study. The renal ultrasound results of patients were not all included because of the large sample size and incomplete data, which might lead to the omission of some factors that would affect hematuria, such as nephrolithiasis, renal tumor, etc. Secondly, due to methodological limitations, false negative results could exist. Another limitation was that the origin of red blood cells in urine could not be identified because of lacking of phase contrast microscopy. Finally, we only analyzed the results of a single urinalysis with definition involving a lower threshold without continuous monitoring and follow-up of children with positive hematuria. While we acknowledge the general definition of hematuria involving a higher threshold, our decision to use a more conservative range aimed to identify even minor occurrences of hematuria that may hold clinical significance, particularly in the context of pediatric microtia patients. So, we will need to conduct a multicenter prospective study with a large number of patients and more reliability data to validate our results and offer more accurate basis for clinical diagnosis, treatment and prognosis.

## Conclusion

Higher incidence of hematuria was observed in female children with microtia and no similar results were obtained in the control group. We suggest that the hematuria can be used as a supplementary test for screening renal congenital malformation in female children with microtia, which will help us to earlier recognition of microtia associated renal anomalies, perform earlier nephrology or urology intervention, and decrease fewer downstream renal complications.

## Data Availability

Data herein reported are fully available in Tables 1, 2, 3, and 4.

## References

[1] Alexander NL, Kini SD, Liu Y-C C (2020). Cardiac anomalies in microtia patients at a tertiary pediatric care center. Int. J. Pediatr. Otorhinolaryngol..

[2] Guo F (2021). Congenital heart defects in patients with isolated microtia: Evaluation using colour doppler echocardiographic image. Cardiol. Young..

[3] González-Andrade F, López-Pulles R, Espín VH, César P-Y-M (2010). High altitude and microtia in Ecuadorian patients. J. Neonatal. Perinatal. Med..

[4] Agopian AJ, Langlois PH, Anushuya R, Canfield MA (2009). Epidemiologic features and clinical subgroups of anotia/microtia in Texas. Birth Defects Res. A Clin. Mol. Teratol..

[5] Suutarla S, Rautio J, Ritvanen A, Ala-Mello S, Jero J, Klockars T (2007). Microtia in Finland: Comparison of characteristics in different populations. Int. J. Pediatr. Otorhinolaryngol..

[6] Forrester MB, Merz RD (2005). Descriptive epidemiology of anotia and microtia, Hawaii, 1986–2002. Congenit Anom (Kyoto)..

[7] Huang X (2021). Evaluation of respiratory system anomalies associated with microtia in a Chinese specialty clinic population. Int. J. Pediatr. Otorhinolaryngol..

[8] Cabrejo R, Persing J, Alperovich M (2019). Epidemiologic assessment of microtia in over 23 million consecutive United States births. J. Craniofac. Surg..

[9] Wang RY, Earl DL, Ruder RO, Graham JM (2001). Syndromic ear anomalies and renal ultrasounds. Multicent. Stud. Pediatr..

[10] Kinia S, Bartonb GW, Liua Y-CC (2020). Renal anomalies and microtia: Determining the clinical utility of screening affected children. Int. J. Pediatr. Otorhinolaryngol..

[11] Jlk A, Ma B, Mmg A, Kwc B, Mai T (2018). Renal ultrasound abnormalities in children with syndromic and nonsyndromic microtia. Int. J. Pediatr. Otorhinolaryngol..

[12] Brent B (1999). The pediatrician's role in caring for patients with congenital microtia and atresia. Pediatr. Pediatr Ann..

[13] Heike CL (2013). Clinical care in craniofacial microsomia: A review of current management recommendations and opportunities to advance research. Am. J. Med. Genet. C Semin. Med. Genet..

[14] Cohen MM, Rollnick BR, Kaye CI (1989). Oculoauriculovertebral spectrum: an updated critique. Cleft Palate J..

[15] Zim S, Lee J, Rubinstein B, Senders C (2017). Prevalence of renal and cervical vertebral anomalies in patients with isolated microtia and/or aural atresia. Cleft Palate Craniofac. J..

[16] Vedula R, Iyengar AA (2020). Approach to diagnosis and management of hematuria. Indian J. Pediatr..

[17] Davis R (2012). Diagnosis, evaluation and follow-up of asymptomatic microhematuria (AMH) in adults: AUA guideline. J. Urol..

[18] Yamauchi M (2012). Clinical and genetic analysis of microtia in Japan. J. Plast. Surg. Hand Surg..

[19] Lee KT (2012). Association of congenital microtia with environmental risk factors in South Korea. Int. J. Pediatr. Otorhinolaryngol..

[20] Luquetti DV, Cox TC, Lopez-Camelo J (2013). Preferential associated anomalies in 818 cases of microtia in South America. Am. J. Med. Genet. A.

[21] van Nunen DP, Kolodzynski MN, van den Boogaard MJ (2014). Microtia in the Netherlands: Clinical characteristics and associated anomalies. Int. J. Pediatr. Otorhinolaryngol..

[22] Stoll C, Alembik Y, Dott B (2016). Associated anomalies in cases with anotia and microtia. Eur. J. Med. Genet..

[23] Beck Jr LH, Salant DJ (2008). Glomerular and tubulointerstitial diseases. Prim. Care Clin. Office Pract..

[24] Kevin ECM (2004). Evaluation of hematuria in children. Urol. Clin. N. Am..

[25] Quigley R (2008). Evaluation of hematuria and proteinuria: How should a pediatrician proceed?. Curr. Opin. Ped..

[26] Vinen C (2003). Oliveira. Acute glomerulonephritis. Postgrad. Med. J..

[27] Youn T, Trachtman H, Gauthier B (2006). Clinical spectrum of gross hematuria in pediatric patients. Clin. Pediatr..

[28] Bignall ONR, Dixon BP (2018). Management of hematuria in children. Curr. Treat. Options Pediatr..

[29] Patil PM, Hipparagi S, Sinha K, Sorangavi V, Patil BM (2014). Asymptomatic proteinuria and hematuria in school going children. J. Krishna Inst. Med. Sci. Univ..

[30] Moghtaderi M (2014). Screening for microscopic hematuria in school-age children of Gorgan CityIran. Iran J. Kidney Dis..

[31] Vivante A (2011). Persistent asymptomatic isolated microscopic hematuria in Israeli adolescents and young adults and risk for end-stage renal disease. J. Am. Med. Assoc..

[32] Cuilan L (2012). The epidemic investigation of asymptomatic hematuria between 0–3years old children in Zhongshan city. Mod. Hosp..

[33] Yanagihara T (2007). Epidemiology of school urinary screening over a 30-year period in Tokyo. Pediatr Int..

[34] Ministry of Education and Human Resources Development. Ducational Statistics System (2002). Sum of results in laboratory tests for school children. Stat. Yearb. Korean Minist. Educ. Hum. Resour. Dev..

[35] Lin CY, Hsieh CC, Chen WP, Yang LY, Wang HH (2001). The underlying diseases and follow-up in Taiwanese children screened by urinalysis. Pediatr. Nephrol..

[36] Murakami M, Hayakawa M, Yanagihara T, Hukunaga Y (2005). Proteinuria screening for children. Kidney Int. Suppl..

[37] Okur M (2013). Determination of underlying causes in asymptomatic, early-stage renal diseases by dipstick test. Med. Glas. (Zenica).

[38] Horie S (2014). Japanese guidelines of the management of hematuria 2013. Clin. Exp. Nephrol..

[39] Phelan PJ, Rheault MN (2018). Hearing loss and renal syndromes. Pediatr. Nephrol..

[40] Kashtan C (2021). Multidisciplinary management of alport syndrome: Current perspectives. J. Multidiscip. Healthc..

[41] Walker KA, Sims-Lucas S, Bates CM (2016). Fibroblast growth factor receptor signaling in kidney and lower urinary tract development. Pediatr. Nephrol..

[42] Brown DD, Reidy KJ (2019). Approach to the child with Hematuria. Pediatr. Clin. North Am..

[43] Zhang Y, Ding J (2018). Renal, auricular, and ocular outcomes of Alport syndrome and their current management. Pediatr. Nephrol..

[44] Miyakita H (2020). Guidelines for the medical management of pediatric vesicoureteral reflux. Int. J. Urol..

[45] Cooper CS, Austin JC (2004). Vesicoureteral reflux: Who benefits from surgery?. Urol. Clin. North Am..

[46] Fidan K, Kandur Y, Buyukkaragoz B, Akdemir UO, Soylemezoglu O (2013). Hypertension in pediatric patients with renal scarring in association with vesicoureteral reflux. Urology.

[47] Zhang W (2020). Relationship between vesicoureteral reflux and glomerular filtration rate in children. Curr. Med. Sci..

[48] Zhong X (2021). Risk factors associated with abnormal urinalysis in children. Front. Pediatr..

